# Supplementary material for: An Optimized FI-RSV Vaccine Effectively Protects Cotton Rats and BALB/c Mice without Causing Enhanced Respiratory Disease
Source: Viruses. 2022 Sep 20;14(10):2085. doi: 10.3390/v14102085 (PMC9612074; doi:10.3390/v14102085)
Supplement: Supplementary file 1 [file viruses-14-02085-s001.zip › viruses-1845964-supplementary.pdf]

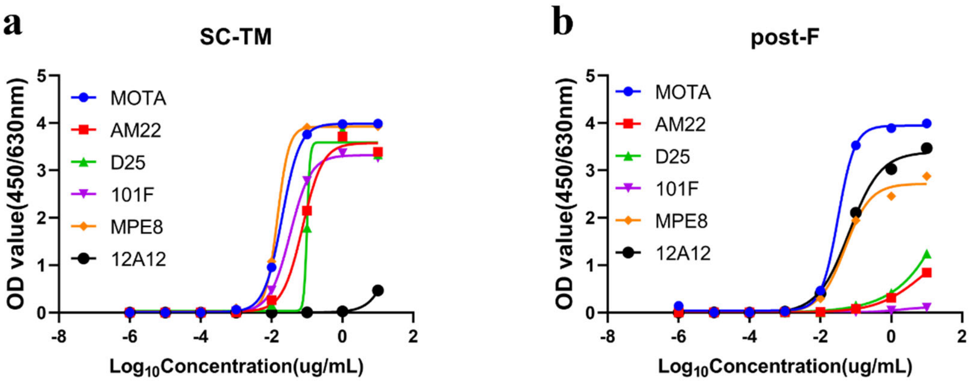

Figure S1. The identity of the prefusion conformation and postfusion conformation on pre-F protein (SC-TM) or post-F protein was verified using ELISA with pre-F specific antibodies (AM22, D25) and total F target mAbs (MPE8, 101F, MOTA) or a post-F-specific mAb (12A12). (**a**) SC-TM. (**b**) post-F.

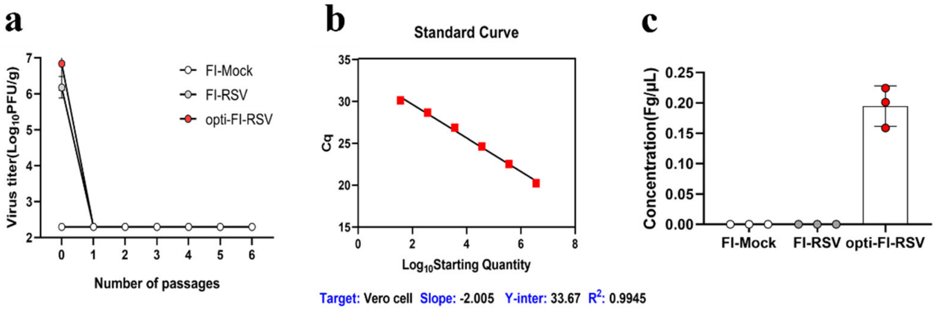

Figure S2. The complete inactivation of vaccines and the levels of residual host cell DNA for vaccines were determined with a plaque assay and a sensitive and specific real-time PCR method, respectively. (**a**) Freshly harvested virions were collected at different time points (for FI-Mock and FI-RSV, 72 hours post-infection; for opti-FI-RSV, 63 hours post-infection) and treated with formalin as described in the Method. The infectivity of both groups was determined before (passages number=0) and post formalin treated and then passaging the material on HEp-2cells for multiple passages. (**b**) The standard curve obtained from standard plasmids analysis. R^2 and Slope were shown. (**c**) Quantification of residual host cell DNA for FI-Mock, FI-RSV and opti-FI-RSV by interpolation of the standard curve. The symbols indicate each independent experiment, the bars/lines indicate the mean ± SD.

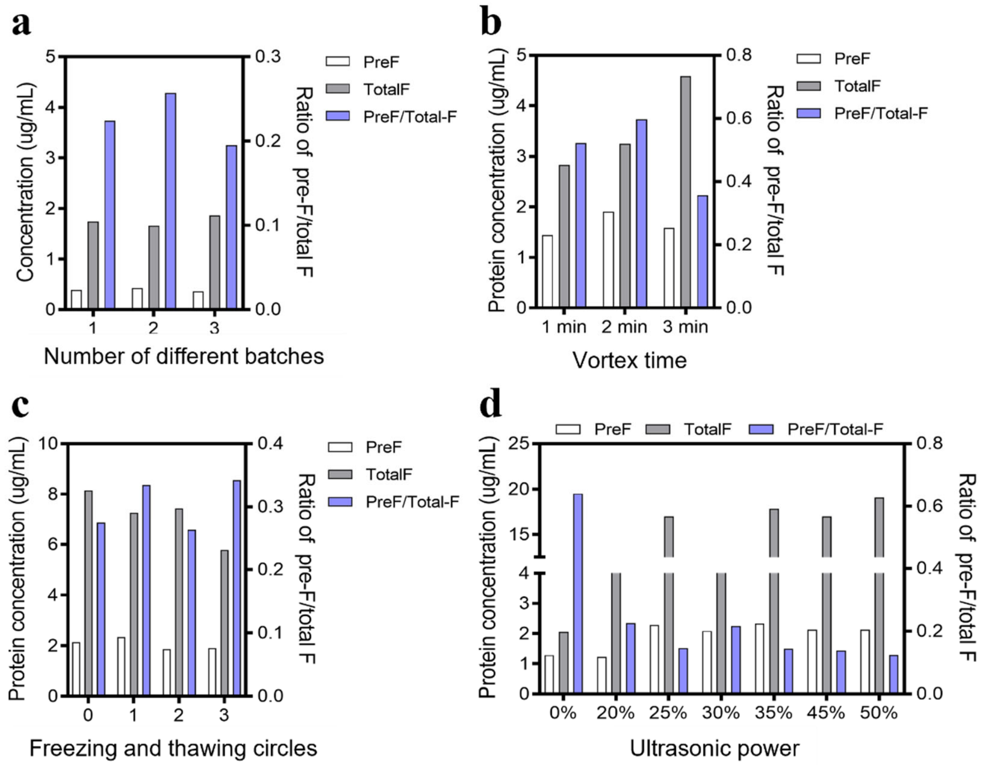

Figure S3. Quantification of pre-F on freshly produced RSV virion stocks under different conditions. (**a**) Vero cells were infected with RSV A2 at MOI=0.3, and the viruses were harvested 72 hours after infection. The harvested viruses were first sonicated at 50% power, followed by centrifugation and supernatant collection. The ratio of the results of sandwich ELISA using AM22, a pre-F-specific antibody, to the results of sandwich ELISA using motavizumab, a total F antibody, is shown. (**b**) Infection was performed as (**a**), except that the viruses were been vortexed rather than ultrasonic. (**c**) Infection was performed as (**a**), and ultrasonic was replaced by freezing and thawing circles. (**d**) Infection was performed as (**a**), and different power of ultrasonic were performed.

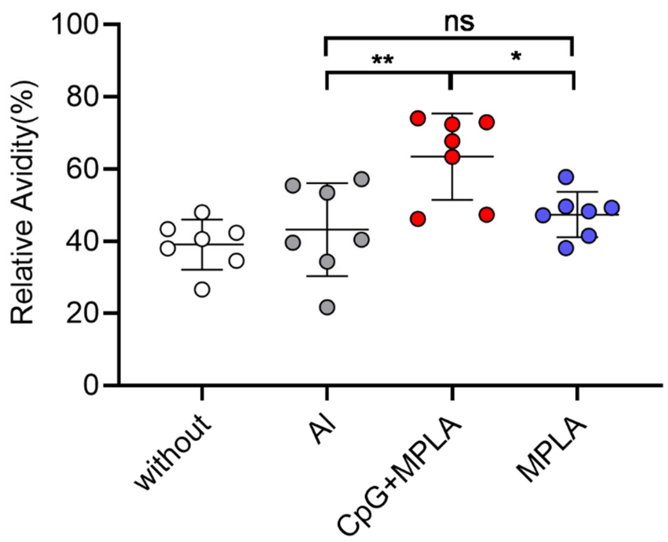

Figure S4. Antibody avidity induced by opti-FI-RSV combined with different adjuvants. Female BALB/c mice (n=7 per group) were injected intramuscularly with opti-FI-RSV combined with different adjuvants in a prime-boost regimen at week 0 and week 3. IgG avidity against pre-F after a 7 M urea wash was determined at 2 weeks after boosting. The symbols indicate individual mice, the bars/lines indicate the mean ± SD. The data were analyzed by one-way ANOVA followed by Tukey's multiple comparisons post hoc test to analyze the differences between vaccination groups. The P values are shown by asterisks (ns=nonsignificant; *P <0.05; **P <0.01; **** P <0.0001).

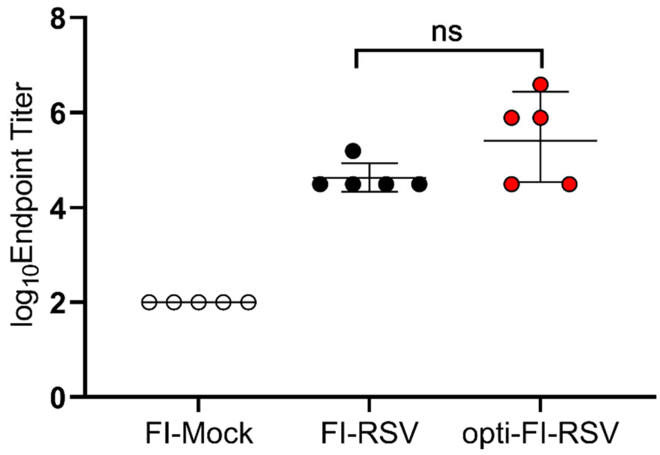

Figure S5. Glycoprotein specific antibodies induced by FI-Mock, FI-RSV and opti-FI-RSV. Female cotton rats (n=5 per group) were injected intramuscularly with FI-Mock, FI-RSV or opti-FI-RSV in a prime-boost regimen at week 0 and week 3. G-specific antibody levels were determined using ELISA. The symbols indicate individual rats, the bars/lines indicate the geometric mean ± geometric SD. The data were analyzed by one-way ANOVA followed by Tukey's multiple comparisons post hoc test to analyze the differences between vaccination groups. The P values are shown by asterisks (ns=nonsignificant; *P <0.05; **P <0.01; **** P <0.0001).

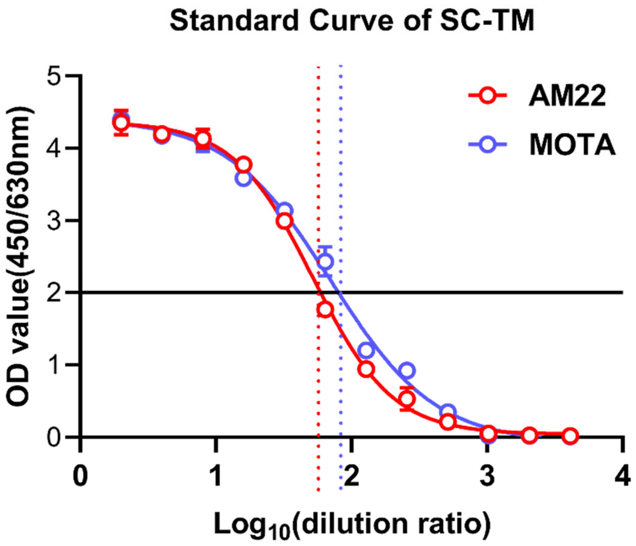

Figure S6. Standard curve for quantitation of F protein by sandwich ELISA. The standard protein SC-TM was diluted to a concentration of 2 µg/mL, and then serially diluted 2-fold. Mixing standards with the diluted biotinylated antibody at a 1:1 ratio. The OD value was measured on a microplate reader. A standard curve was obtained by plotting the mean absorbance values against the logarithm-transformed SC-TM concentrations and fitted to a four-parameter logistic equation using GraphPad Prism. The symbols indicate experiment in triplicates, the bars/lines indicate the mean ± SD.
